# Synthesis of Hollow Sphere and 1D Structural Materials by Sol-Gel Process

**DOI:** 10.3390/ma10090995

**Published:** 2017-08-25

**Authors:** Fa-Liang Li, Hai-Jun Zhang

**Affiliations:** 1 The State Key Laboratory of Refractories and Metallurgy, Wuhan University of Science and Technology, Wuhan 430081, China; zhanghaijun@wust.edu.cn; 2 Jiangxi Engineering Research Center of Industrial Ceramics, Pingxiang 337022, China

**Keywords:** sol-gel, hollow sphere, 1D structure

## Abstract

The sol-gel method is a simple and facile wet chemical process for fabricating advanced materials with high homogeneity, high purity, and excellent chemical reactivity at a relatively low temperature. By adjusting the processing parameters, the sol-gel technique can be used to prepare hollow sphere and 1D structural materials that exhibit a wide application in the fields of catalyst, drug or gene carriers, photoactive, sensors and Li-ion batteries. This feature article reviewed the development of the preparation of hollow sphere and 1D structural materials using the sol-gel method. The effects of calcination temperature, soaking time, pH value, surfactant, etc., on the preparation of hollow sphere and 1D structural materials were summarized, and their formation mechanisms were generalized. Finally, possible future research directions of the sol-gel technique were outlined.

## 1. Introduction

The sol-gel method has attracted much attention as it can be used to fabricate high purity products with a fine particle size and good chemical homogeneity at low temperatures. With outstanding advantages of accuracy, stability, low reaction temperature, and a high purity of targeted products, the sol-gel process is considered as one of synthetic material methods with the most potential.

In the past few decades, the sol-gel method (combined with other techniques including microwave heating, ultrasonication, spin-coating, dip coating, laminar flow coating, and strong field induction) have been used to prepare various kinds of advanced materials with different morphologies including magnetic [1,2,3,4,5,6,7,8,9,10], optical [11,12,13], electronic [14,15], and structural [16,17,18,19,20,21,22,23,24,25] particles, and hollow spheres [26], fibers [27], nanowires [28] and films [29,30,31,32,33,34]. Many reviews have been published on the preparation of particles and coatings using the sol-gel process; for instance, Guo et al. [35] summarized the sol-gel synthesis and application of monodisperse nanoparticles and granules including Li_2_TiO_3_, ZnO-B_2_O_3_-SiO_2_ additive, La_1−x_Sr_x_CoO_3-δ_ (x = 0.1 − 0.7) and Y_3_Al_5_O_12_. Zhang et al. [36] outlined the sol-gel process synthesis of high-temperature non-oxide ultrafine powders including nitride, carbide and boride. This demonstrated that the sol-gel process offered many advantages in the preparation of powders, such as lower formation temperatures, shorter soaking times, and the ability to synthesize submicron and nano-crystalline ultrafine powders. Moreover, the resultant powders possessed a narrow and uniform distribution, higher homogeneity and purity. Meanwhile, the corresponding electronic ceramics prepared by the as-prepared nanopowders could be sintered at a lower temperature, and showed good temperature stability and high electrical properties. This also indicated that the process suffered several drawbacks including the requirement of expensive precursors and additives, and it was difficult to control the structure of the targeted powders. On the other hand, Guo et al. [35] summarized the sol-gel preparation of many types of functional coatings, including anti-reflection, anticorrosion, wearable, and anti-soiling coatings. Livage et al. [37] reviewed sol-gel electrochromic coatings including WO_3_, MoO_3_, V_2_O_5_, Nb_2_O_5_, TiO_2_, CeO_2_, IrO_2_, Fe_2_O_3_, and NiO. Yoldas [38] pointed out that sol-gel coatings could be used on optical fibers and photovoltaic cells, as well as demonstrated that sol-gel coatings offered many excellent properties such as easy doping, low cost, no shape limitation, good adhesion, and crack-free.

Recently, studies have been carried out to prepare hollow spheres and 1D structural materials as it is well-known that hollow spheres can be widely used in the fields of therapeutics, energy storage, electronics, environmental remediation, medical ultrasounds, biosensors, non-destructive testing, electronic devices, and low-density transducer arrays given their excellent properties of low density, high specific surface area, high energy conversion efficiency, high adsorption capacity, and large light-harvesting efficiencies [39,40,41,42,43,44,45,46,47,48,49,50,51]. Furthermore, 1D structural materials such as fibers, nanorods, and nanowires have attracted a lot of attention in the areas of catalysis, reinforcement, sensors, solar cells, super-capacitors, optics, electronics, etc., due to their unique properties such as light volume-weight, high strength and modulus, high thermo-mechanical stability, and enhanced photocatalytic activity [52,53,54,55,56,57,58,59,60,61,62,63].

In this review, we provide an overview of recent developments in the fabrication of hollow sphere and 1D structural materials using the sol-gel technique. Finally, probable improvements and future outlooks of the sol-gel method are outlined.

## 2. Synthesis of Hollow Sphere Materials by the Sol-Gel Method

The template method (including hard template and soft template methods) is considered as the most effective route for preparing hollow structures. Various kinds of hollow spheres have been prepared by the sol-gel technique based on the template method.

**Figure 1:**
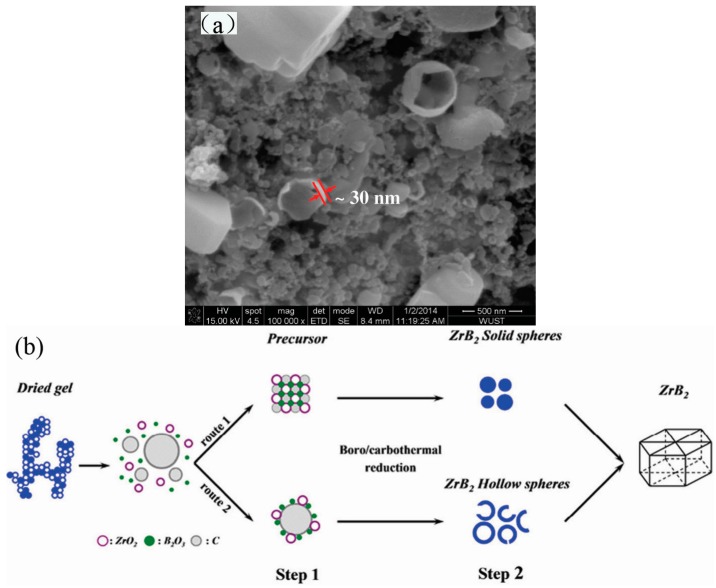
(**a**) SEM image of ZrB_2_ prepared at 1500 °C for 2 h; (**b**) Schematic illustration of the grown process of hollow ZrB_2_ spheres crystals [65].

Binary metal borides exhibit excellent mechanical properties. Among them, zirconium diboride (ZrB_2_) is seen as one of the most promising ultra-high temperature ceramics owing to its high melting point (3040 °C), high hardness (22 GPa), good chemical stability, good corrosion resistance, and good thermal shock resistance [64]. Our group successfully synthesized ZrB_2_ ultrafine hollow spherical powders using sol-gel combined with a boro/carbothermal reduction method using zirconium oxychloride, boric acid, and glucose as the main raw materials [65]. The effects of reaction temperature, amount of B, Zr, and C on the formation of ZrB_2_ were studied and indicated that ZrB_2_ could be synthesized at 1200 °C, and that ZrB_2_ hollow spheres were successfully prepared at 1500 °C for 2 h when the molar ratios of B/Zr = 2.5 and C/Zr = 6.5. The formation temperature was about 300 °C lower than that demanded by the conventional solid-state mixing method [32]. Scanning electron microscopy SEM results showed that the diameters of ZrB_2_ ultrafine hollow spheres ranged from 100 to 500 nm with an average shell thickness of about 30 nm (Figure 1a). The possible mechanism for the formation of ZrB_2_ ultrafine hollow spheres is illustrated in Figure 1b. First, large carbonaceous spheres with sizes ranging from 100 to 500 nm were formed during the carbonization of glucose and citric acid. Second, the as-formed carbonaceous spheres attached to ZrO_2_/B_2_O_3_ precursors on their surfaces. Finally, when the gel was heated at high temperature, the oxide precursors reacted with the carbonaceous sphere templates to form in situ ZrB_2_ hollow spheres. The results also indicated a low yield of hollow spheres via this method.

**Figure 2:**
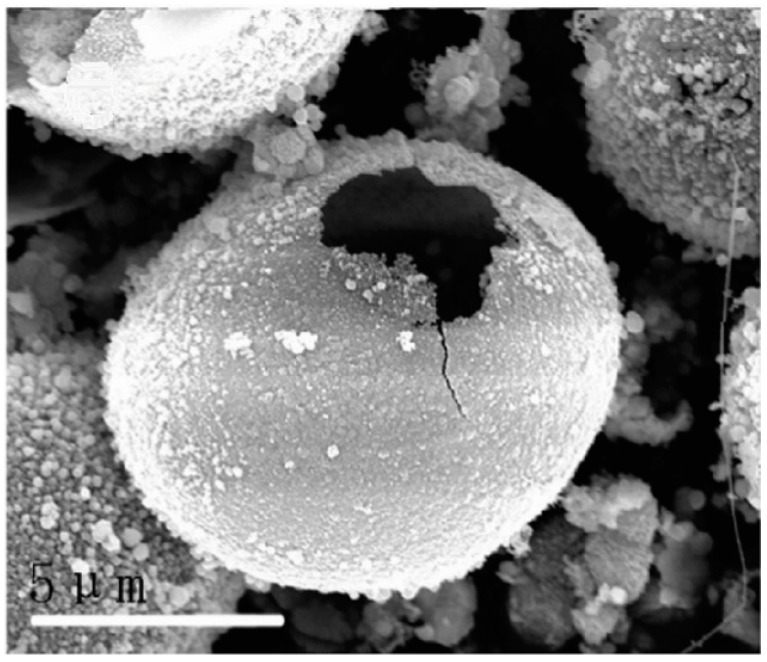
SEM image of as-prepared *β*-SiC [66].

Given the outstanding properties of high strength, high thermal conductivity, high chemical stability, wide band gap energy (~2.4 eV), low negative conduction band potential (−1.40 V), and good environmental friendliness, silicon carbide (SiC) has been widely used from metallurgy to aerospace. Wang et al. [66] successfully fabricated openmouthed *β*-SiC hollow spheres through the environmentally friendly sol-gel method using glucose as a carbon resource and poly(ethylene oxide)–poly(propylene oxide)–poly(ethylene oxide) (PEO–PPO–PEO) as a silicon source, and the effects of carbothermal reduction temperature and time on the synthesis of *β*-SiC were investigated. They indicated that the preparation of *β*-SiC hollow spheres was significantly affected by the calcination temperature and time. When the precursors were fired at 1450 °C for 8 h, the as-prepared SiC samples exhibited openmouthed hollow microsphere morphology with sizes of about 10 μm. When the calcination temperature was increased to 1500 °C, no whole hollow microspheres were obtained. The fabrication of *β*-SiC hollow spheres was suggested through the following five stages: (1) The PEO-PPO-PEO and glucose were wrapped together to form a microsphere template, and the SiO_2_ particles derived from the hydrolysis of tetraethyl orthosilicate were self-assembled onto the template spherical surface; (2) Heating the PEO-PPO-PEO and glucose microspheres at 550 °C under an N_2_ atmosphere to form carbon spheres; (3) SiC sphere shells were produced via the reaction between the outer SiO_2_ and carbon sphere core by increasing the heating temperature to 1450 °C; (4) The unreacted carbon core was burned out when the samples were calcined at 600 °C in an O_2_ atmosphere, which resulted in an open mouth on the shell of SiC sphere; and (5) Unreacted SiO_2_ was removed using an NaOH solution, and finally openmouthed *β*-SiC hollow spheres with rough surface were obtained (Figure 2). The UV-vis spectra revealed that the as-prepared *β*-SiC hollow spheres displayed a blue shift of absorption edges, which was caused by the little change in crystal structure of SiC with calcination temperature and time. Furthermore, the prepared *β*-SiC products could photocatalytically reduce CO_2_ to CH_4_ with better efficiency than the standard photocatalyst P25 TiO_2_. It is believed that one reason was the very negative conduction band potential of −1.40 V of the resulting *β*-SiC products, and the other reason was their special morphology. The special hollow sphere structure brought a higher BET surface area and allowed multi-reflections of illumination light within its interior cavities, which was an efficient use of the light source and finally enhanced photocatalytic activity.

**Figure 3:**
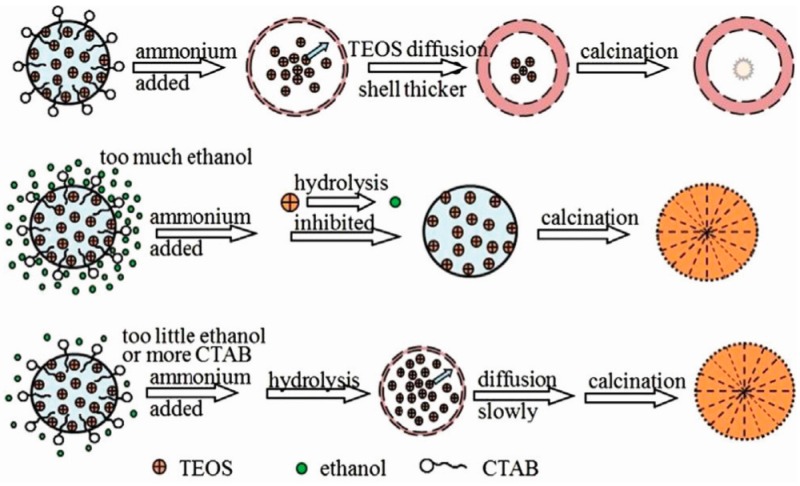
Schematic illustrations for the controllable synthesis of silica hollow sphere [67].

Hollow mesoporous silica spheres which have been widely used in the fields of delivery, separation and catalysis can be also synthesized through the sol-gel method. Wang et al. [67] synthesized hollow mesoporous silica spheres using the sol-gel/emulsion method with cetyltrimethylammonium bromide (CTAB) as the surfactant to stabilize the tetraethoxysilane (TEOS) droplet, and the effects of ethanol on the formation of silica hollow spheres were studied. Transmission electron microscopy (TEM) images revealed that hollow silica spheres could form at an ethanol to water ratio from 0.62 to 0.47, and that the wall thickness of the hollow silica spheres decreased with decreasing ethanol content. Additionally, the HR-TEM images showed that mesopores radiated throughout the spheres and that CTAB concentration played an important role on the synthesis of hollow mesoporous silica spheres. Interfacial energy was reduced by introducing more CTAB into the system that resulted in smaller TEOS droplets, which were enclosed by CTAB micelles that served as templates for the formation of silica hollow spheres. By adding a solution of aqueous ammonia, TEOS on the interface was hydrolyzed into silica and then deposited onto the interface. Along with a decrease of the concentration of TEOS on the interface, the TEOS would also diffuse from the inside to the interface for concentration equilibrium. Finally, hollow silica spheres were obtained after calcination. The proposed schematic of the formation of silica spheres is shown in Figure 3. It also indicates that ethanol content is crucial for the formation of SiO_2_ hollow spheres. If too little ethanol was introduced into the system, the hydrolysis speed of TEOS was faster than their diffusion speed in the droplets. As a result, the synthesized silica spheres were all solid, and the resultant hollow mesoporous silica spheres could be used as catalyst support. 

**Figure 4:**
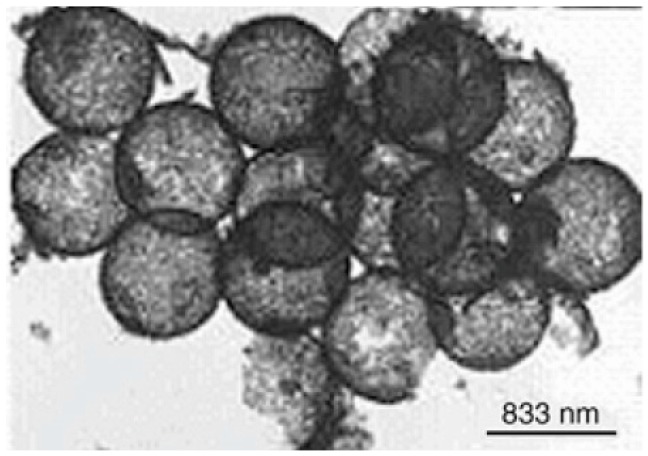
Transmission electron microscopy (TEM) image of Eu_2_O_3_ hollow sphere [68].

Rare earth oxide hollow spheres can also be prepared with the sol-gel method. Europium sesquioxide (Eu_2_O_3_) is widely used in scintillators, catalysis, electrochemical energy-storage devices, and luminescent materials. Zhang et al. [68] successfully synthesized Eu_2_O_3_ hollow spheres via a sol-gel method using polystyrene/polyelectrolyte microspheres as the templates. The X-ray diffraction XRD results indicated that phase pure cubic structured Eu_2_O_3_ was obtained when the precursors were fired at 700 °C for 3 h. TEM and FFSEM images showed that the shell thickness and outer diameter of the as-prepared Eu_2_O_3_ hollow spheres was about 75 and 690 nm, respectively (Figure 4). The mechanism of the formation of hollow sphere Eu_2_O_3_ was also proposed as follows: (1) when the polystyrene/polyelectrolyte templates were dipped into the mixed solution (containing europium nitrate and urea), the pores of the templates were easier to fill with the solution owing to the lower viscosity; (2) in the next heating treatment, urea was hydrolyzed to form OH^−^ ions that increased the pH value of the solution, and the OH^−^ ions reacted with europium to form a Eu[(OH)_3_](H_2_O)_y_ sol, which took place simultaneously within the pores and the solution; and (3) during the calcination process, the sol is transformed to gel by the condensation reactions within the polyelectrolyte microreactor. Meanwhile, the polystyrene/polyelectrolyte template was removed and finally formed a hollow sphere structure. Luminescence spectra demonstrated that the excitation wavelength of the resultant Eu_2_O_3_ hollow sphere was 514.5 nm at room temperature. Compared with bulk Eu_2_O_3_, the peak in luminescence spectra of the obtained sample was obviously broadened.

The sol-gel method can also be used to prepare srilankite-type zirconium titanate (ZrTiO_4_), which has been widely used in microwave telecommunications, the manufacture of high temperature pigments, catalysis, and photocatalysis. Syoufian et al. [69] successfully synthesized ZrTiO_4_ hollow spheres of a submicrometer size via the sol-gel method using sulfonated polystyrene latex particles as the template. TEM images indicated the hollow structure of the resultant ZrTiO_4_ spheres and revealed that the spherical shell consisted of a dense arrangement of ZrTiO_4_ nano-crystals with relatively smooth surfaces. The average outer and void diameter of ZrTiO_4_ hollow spheres was approximately 190 nm and 160 nm, respectively. The homogeneous spherical shell was determined by the amphiphilic nature of the sulfonated polystyrene latex particles which allowed them to be easily distributed in the solvent, and provided suitable sites for the attachment of tetrabutyl titanate and tetrabutyl zirconate. UV-vis absorption spectra revealed that the band gap energy *E_g_* of ZrTiO_4_ hollow spheres was higher than that of the TiO_2_ powders (Degussa P25), which illustrated that their potential redox in the photocatalytic system also increased. This may be attributed to the following two factors: (1) The smaller and denser building blocks of the hollow sphere shell wall, whose special structure led to the blue-shift in absorption spectra because of the quantum size effect; and (2) the existence of Zr (as ZrO_2_) within the TiO_2_ framework, which would enhance the UV-responsiveness as ZrO_2_ is a direct band gap semiconductor. Therefore, the prepared ZrTiO_4_ hollow spheres are a promising photocatalyst candidate with higher redox potential. 

As one of the most promising piezoelectric materials, lead zirconate titanate (PZT) has excellent properties such as large electromechanical coupling coefficients, high resistance to depolarization, and high temperature stability. Yang et al. [70] successfully fabricated PZT hollow spheres by using the sol-gel method where polyacrylamide latex solid microspheres and PZT sol were prepared before the polyacrylamide spheres were poured into the PZT sol. The PZT sol was gelled inside the solid polyacrylamide spheres. After that, the gel precursors were fired at high temperatures to obtain hollow sphere PZT. SEM images revealed that the outer diameter and wall thickness of the resultant PZT hollow sphere was respectively about 1–2 mm and 100 μm. According to the SEM images of the outer surface, small cracks and pores were observed on the surface of the sphere and the grain size on the surface was about 0.8 μm. In contrast, the SEM images of the inner surface showed that the prepared PZT hollow sphere exhibited a rough inner surface owing to non-uniform penetration of the PZT sol into the polyacrylamide sphere. Additionally, rib-like structures were also found on the inner surface, which provided strength to the hollow PZT spheres. It was noted that the density of resultant PZT hollow spheres and the hollow spheres wall was 1.123 and 3.10 g·cm^−3^, respectively, suggesting that not only were the prepared PZT spheres hollow, but that the hollow spheres wall also exhibited a porous structure considering the theoretical density of PZT (about 8.0 g·cm^−3^). Moreover, the planar coupling factor of resultant PZT hollow spheres was lower than that of the dense PZT discs. Therefore, the PZT hollow spheres can be used as light-weight transducers in medical ultrasonics and underwater applications.

**Figure 5:**
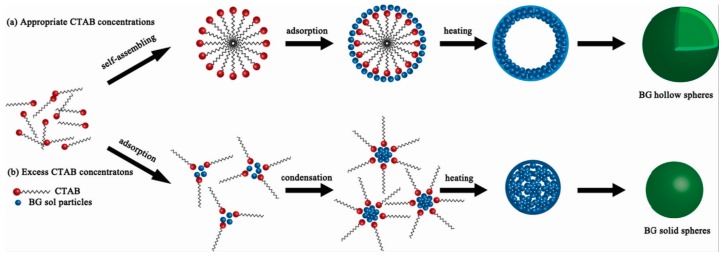
Schematic illustration of the formation processes of hollow mesoporous bioactive glass sub-micron spheres (HMBGS) [71].

The sol-gel method has also been used to prepare bioactive glasses, which have been widely used for bone tissue regeneration due to their good bioactive, resorbable, and osteo-productive properties. Hu et al. [71] successfully fabricated hollow mesoporous bioactive glass sub-micron spheres (HMBGS) via the sol-gel technique using CTAB as a template agent. SEM (combined with the TEM images) revealed that the microstructure of HMBGS was significantly affected by the concentration of CTAB. When introducing 3.3 mM CTAB, the average particle diameter and shell thickness of as-prepared HMBGS was 294 and 32 nm, respectively. With an increase in CTAB concentration from 3.3 to 5.9 mM, the spheres became solid with an average particle diameter of 87 nm. The N_2_ absorption–desorption isotherm results showed that the specific surface area of the prepared HMBGS was larger than 444.0 m^2^·g^−1^ and the average pore size of the prepared HMBGS was larger than 4.6 nm. This indicated that the formation of hollow mesoporous structures was dominated by a surfactant-template mechanism. As shown in Figure 5, CTAB self-assembled into spherical vesicles in the ethanol-water solution after stirring first. With increasing CTAB concentration in the system, the hydrolysis of TEOS was accelerated. As a result, the nucleation rate was faster than the CTAB self-assembling rate, and a large number of bioactive glass sol particles were formed. Second, the CTAB molecules adsorbed to the surface of the sol particles by hydrogen bonding interactions. Finally, CTAB was removed by calcination at high temperature and solid mesoporous bioactive glass spheres were obtained. In contrast, if the CTAB self-assembling rate was faster than the nucleation rate, the CTAB molecules self-assembled into spherical vesicles. Next, the bioactive glass sols were adsorbed onto the surface of the vesicle by hydrogen bonding interactions. Finally, HMBGS were obtained after the removal of CTAB through high temperature treatment. The prepared HMBGS can be used as good candidates for drug or gene carriers in bone tissue regeneration.

**Figure 6:**
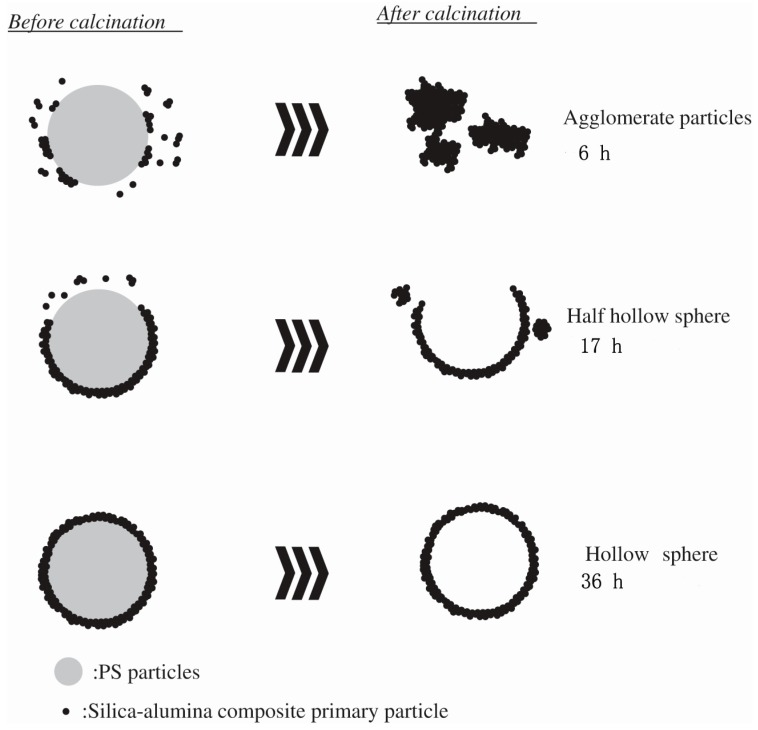
Schematic illustration of formation process of the hollow spheres with different coating time (6, 17, 36 h) [72].

Not only single phase hollow spheres, but also composite hollow sphere materials can be prepared by the sol-gel method. Toyama et al. [72] prepared hollow silica-alumina (SiO_2_-Al_2_O_3_) composite spheres via the sol-gel method using polystyrene particles as a template. Methanol, ethanol, and 2-propanol were separately used as solvents to investigate the effect of alcohol solvents on the morphology of hollow SiO_2_-Al_2_O_3_ composite spheres. When methanol was used as a solvent, only aggregate particles formed after 6 h. By increasing the coating time to 17 h, half hollow spheres and hollow spheres with diameters of approximately 260 nm were observed in the samples. If the coating time was allowed to continue to 36 h, hollow spherical particles with diameters of about 260 nm were formed. The formation of hollow spheres prepared by the polystyrene template method is schematically illustrated in Figure 6. First, the positively charged polystyrene particles were prepared via emulsion polymerization using azo diisobutyl amidine hydrochloride as the initiator, and the silica-alumina composite primary particles with a negative charge were then prepared in a basic solution. Next, the silica-alumina composite primary particles were attracted to the polystyrene templates through electrostatic interaction, and the silica-alumina composite primary particles were sparsely coated on polystyrene template particles to form composite shells. Finally, the polystyrene template was burned out at high temperature and hollow SiO_2_-Al_2_O_3_ composite spheres were obtained. This showed that the amount and rate of hydrogen evolution of the prepared hollow spheres were dependent on the solvent. When methanol or ethanol was used as the solvent, the as-prepared hollow SiO_2_-Al_2_O_3_ composite spheres showed a higher hydrolytic dehydrogenation of NH_3_BH_3_. 

Aside from the above-mentioned hollow spheres, other hollow sphere materials such as Pt-doped TiO_2_ hollow spheres [73], organosilica spheres [74], Fe/CeO_2_ hollow spheres [75], SrTiO_3_ hollow spheres [76], etc. have also been fabricated by the sol-gel technique. According to the work that has been reported, it can be seen that both oxide and non-oxide hollow sphere materials can be fabricated at relatively low temperatures and short duration times by using the sol-gel technique based on template method. The size, shell thickness, and porosity of the hollow spheres can be tailored by controlling the processing parameters of the method. Additionally, the prepared hollow sphere products always showed a relatively narrow particle size distribution and good dispersibility. Therefore, both single phase and composite hollow sphere materials can be fabricated by the sol-gel method. However, it should be noted that there are still several drawbacks to overcome to prepare hollow spheres using the present sol-gel process. For example, cracks or pores were always found on the surface of the hollow spheres due to the escaping of organic materials, the yield of hollow spheres was low and a long gelling time was usually required, and the solvent used to remove the template was often harmful. Therefore, a new process should be developed for improving product yield. On the other hand, the contents of organic matter should be decreased to reduce defects in the targeted products. 

## 3. Synthesis of 1D Structural Materials by the Sol-Gel Method

1D structural materials can be prepared by various techniques such as template, electrolysis, sol-gel synthesis, hydrothermal growth, and viscous solution spinning [77]. Up to now, different kinds of 1D structural materials have been prepared by the sol-gel based method.

**Figure 7:**
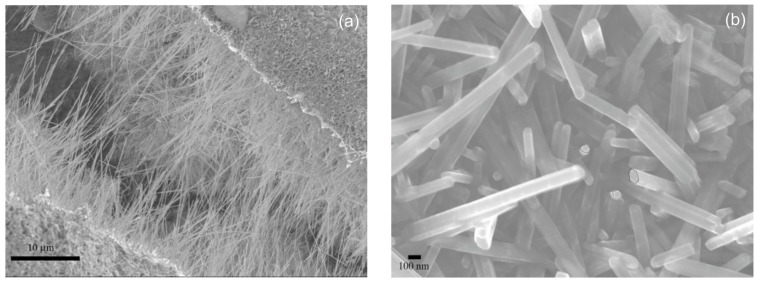
SEM images of the MgB_2_ nanowires. (**a**) A thick mesh of nanowires; (**b**) Higher-magnification image of the vertically oriented nanowires [79].

Magnesium boride (MgB_2_) is a well-known superconductor material with a relative transition temperature of approximately −234 °C [78]. Nath et al. [79] prepared MgB_2_ nanowires by a simple sol-gel method with magnesium bromide and sodium borohydride as the main raw materials. First, a precursor gel was synthesized by mixing magnesium bromide and sodium borohydride reagents in the presence of CTAB. Then, the resulted gel was pyrolized under a diborane-N_2_ atmosphere for forming MgB_2_. SEM images showed that the as-prepared MgB_2_ nanowires with very smooth surfaces were at least 20 μm in length and about 50–100 nm in diameter (Figure 7). TEM images showed that the synthesized MgB_2_ nanowires were solid, straight, and very uniform in diameter along their lengths. Some nanowires had a rounded tip, while others exhibited a flat rectangular or polygonal tip. The selected area electron diffraction (SAED) patterns of some individual nanowires demonstrated that the nanowires were crystalline. The formation of nanowires was mainly determined by the gel in the initial step, and the prearrangement of the precursor particles could template the formation of the one-dimensional morphology of the nanowires with the participation of a CTAB surfactant. Additionally, B_2_H_6_ gas played an important role in the synthesis process. First, B_2_H_6_ gas can be used to maintain a B-rich atmosphere. Second, B_2_H_6_ reacts with even minute amounts of O_2_ to form solid B-oxide phase and prevent oxygen from making contact with the reactants. The magnetic susceptibility data revealed that the nanowires morphology did not affect the transition temperature, and the synthesized MgB_2_ nanowires exhibited a superconducting temperature of about −234.4 °C, which was close to that of bulk MgB_2_ (−234 °C). Magnetization vs. field measurement demonstrated that MgB_2_ was a type II superconductor.

The sol-gel technique has also been used to prepare SiBON fibers, which can be used as spacecraft material with good wavetransparent and mechanical properties. Li et al. [80] successfully synthesized SiBON fibers through the sol-gel method with boric acid, melamine, and TEOS as raw materials. The SiBON fiber precursor was first prepared via the sol-gel technique and then nitrided at 1400–1800 °C in N_2_ to obtain SiBON fibers, and the effects of reaction temperature and pH value on the fabrication of SiBON fibers were investigated. XRD patterns showed that the resultant SiBON fiber was almost amorphous when the precursor was nitrided at 1400 °C and the crystallization of SiBON fiber was improved with increasing heating temperature. The morphologies of the SiBON fiber were affected significantly by pH value; when pH = 8, the SiBON fiber had a shorter and more uniform distribution when compared with the precursor fiber. After being nitrided, the resultant SiBON fiber became looser and bifurcated at both ends with even thickness when pH = 6. With decreasing pH value to 4, the aspect ratio of fibers (after being nitride) decreased, while the surface turned rough. Fourier transform infrared spectroscopy (FTIR) results revealed that the resultant SiBON fiber had B-N-Si and B-O-Si bonds. It was suggested that the SiBON fiber precursor growth underwent different reactions. First, Si-O and B-O groups were generated in a mixture of TEOS and boric acid. When melamine was added, the O of Si-O and B-O groups was generated by the reaction in solution. As the N of -NH_2_ groups of melamine are strong negative centers, it is easy to form B-N-Si chains. Second, the etherification and dehydration of Si-OH groups derived from the hydrolysis of TEOS formed Si-O-Si chain segments. Si-O-Si chains grew along the SiBON radial direction and eventually formed SiBON fiber precursors with a certain aspect ratio. 

**Figure 8:**
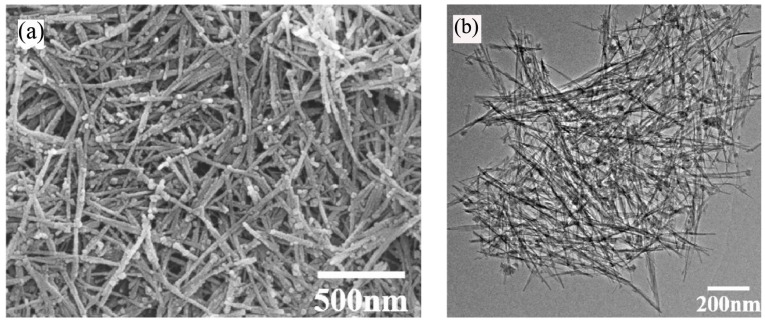
(**a**) SEM image and (**b**) TEM image of the cetyltrimethylammonium bromide (CTAB)-derived MnO_2_ nanowires [81].

Manganese oxides have been used in catalysts, absorbents, and Li^+^ related batteries as they have outstanding structural flexibility and a multitude of oxidation states (Mn^2+^, Mn^3+^, Mn^4+^). Tang et al. [81] prepared ultrafine MnO_2_ nanowires and nanorods via the sol-gel method with different surfactants in an ethanol solvent. Four different surfactants including CTAB, polyvinyl pyrrolidone (PVP, K30), pluronic P123 triblock copolymer (EO_20_PO_70_EO_20_), and sodium dodecyl sulfate were used in the process, and the effects of the surfactants on the fabrication of MnO_2_ nanowires and nanorods were studied. SEM and TEM images showed that products with lengths up to several micrometers consisted of a large amount of highly dispersed ultrafine wire-like structures, which had a diameter of 7 nm (Figure 8). SEM images also revealed that the P123-derived products possessed irregular particle structures, which were aggregated by a large number of nanowires. Based on higher magnification images, nanowires were found with diameters of about 10 nm and with lengths of about 200 nm. A HRTEM image revealed that the interplanar spacing of the lattice planes was about 0.24 nm, which can be ascribed to the (211) crystal planes of the tetragonal MnO_2_. By adding PVP as a surfactant, the obtained XRD patterns were similar to those of the P123-derived products. When sodium dodecyl sulfate was used as the surfactant, SEM images showed that the as-prepared particles were aggregated with a free-standing sheet structure. TEM images revealed that the sheet-like structures consisted of several nanorods with diameters of about 10 nm and lengths of approximately 50 nm. Therefore, the structure of the products was sharply determined by the surfactants.

**Figure 9:**
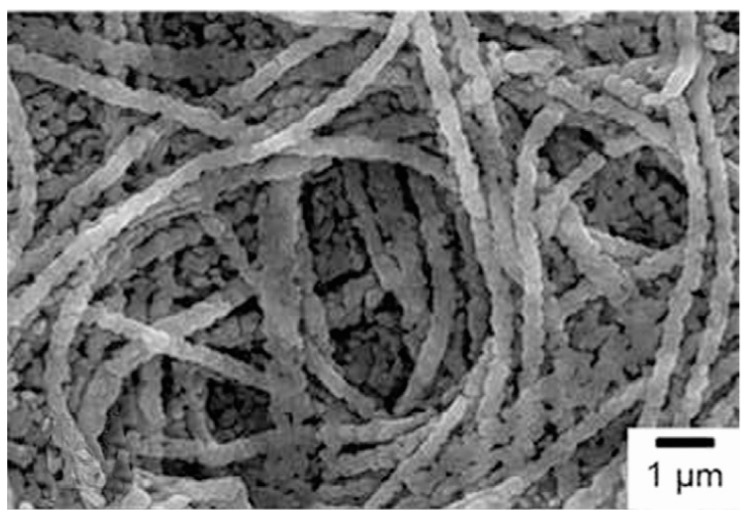
SEM image of MnTiO_3_ fibers calcined at 800 °C [83].

Manganese titanate (MnTiO_3_) has been regarded as a promising material in solar energy systems with strong absorption in the visible region [82]. Nakhowong et al. [83] successfully fabricated MnTiO_3_ nanofibers using the sol-gel assisted electrospinning method with polyvinylacetate, manganese acetate, and titanium (IV) isopropoxide as the main raw materials, and the effect of temperature on the synthesis of MnTiO_3_ nanofibers was also investigated. SEM images showed that the microstructure of the prepared MnTiO_3_ was significantly affected by the calcination temperature. Before heat treatment, the composite fiber precursors exhibited a smooth surface with an average diameter of about 850 nm. When the precursors were heated at 800 °C, the resultant fibers shrunk to an average diameter of about 328 nm with a rough surface (Figure 9). This phenomenon was caused by the decomposition of polyvinylacetate and subsequent crystallization. Increasing the calcination temperature to 900 °C, the obtained MnTiO_3_ nanofibers became discrete in length and the average diameter increased to 415 nm. By increasing the calcination temperature to 1000 °C, only MnTiO_3_ particles were observed in the resultant sample. The FTIR spectrum of prepared MnTiO_3_ nanofibers revealed that the absorption peaks were found at 452 cm^−1^ and 532 cm^−1^, which was associated with Ti-O and Mn-O bands, respectively, and demonstrated the formation of MnTiO_3_ crystalline.

Spinel structure materials also can be prepared by the sol-gel method. ZnMn_2_O_4_ has been widely used in Li-ion batteries, supercapacitors, sensors, and thermistors. Shamitha et al. successfully fabricated ZnMn_2_O_4_ nanofibers via the sol-gel assisted electrospinning method using poly(styrene-*co*-acrylonitrile) as a sacrificial polymeric binder, and the influence of calcination temperature on the synthesis of ZnMn_2_O_4_ nanofibers was investigated [84]. Before heat treatment, the average diameter of the composite fiber precursors was about 281 nm. When the precursors were fired at high temperature, the surface of the resultant fibers became rough and the average diameter of the resultant fibers obviously decreased, which was caused by the elimination of the organic phases. When the calcination temperature increased from 500 to 700 °C, the average diameter of the resultant fibers decreased from 243 to 181 nm due to crystallite growth and densification. The SAED patterns confirmed the formation of ZnMn_2_O_4_ nanofibers and demonstrated that the crystallinity of ZnMn_2_O_4_ nanofibers were enhanced with increasing calcination temperature. The nitrogen adsorption-desorption isotherms revealed that the prepared ZnMn_2_O_4_ nanofibers were mesoporous, which was caused by the elimination of a styrene-acrylonitrile random copolymer and the decomposition of metal acetates during heat treatment. The highest surface area of the prepared ZnMn_2_O_4_ nanofibers was about 79.51 m^2^·g^−1^, which was higher than that reported elsewhere. The reactions that may have occurred during the whole process were as follows:

MnCO_3_ → MnO + CO_2_ (1)

Mn_3_O_4_ + CO → 3MnO + CO_2_ (2)

Mn_2_O_3_ + CO → 2MnO + CO_2_ (3)

MnO_2_ + CO_2_ → MnO + CO_2_ (4)

2ZnO + 2MnO+O_2_ → 2ZnMnO_3_ (5)

ZnMnO_3_ + MnO → ZnMn_2_O_4_ (6)

It was noteworthy that the prepared ZnMn_2_O_4_ nanofibers could be a better electrode material for lithium ion batteries with a superior surface area.

**Figure 10:**
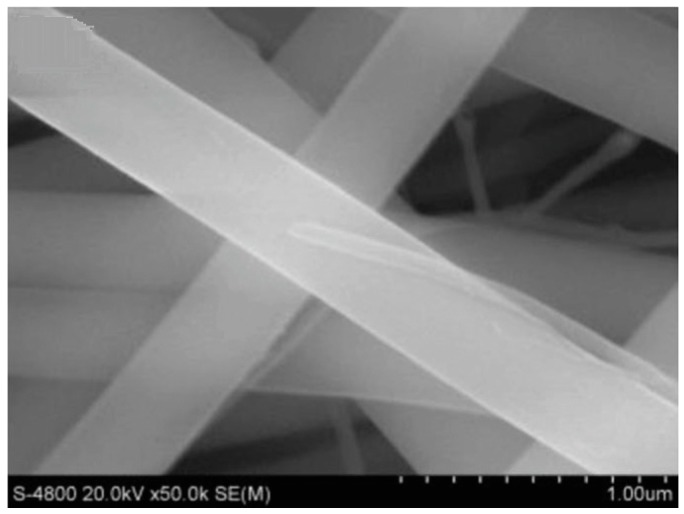
SEM image of the electrospun mullite fibers after calcination at 800 °C [85].

The sol-gel method can be also applied to fabricate mullite fibers, which have exhibited good chemical and thermal stability, excellent high temperature mechanical strength, low thermal expansion coefficient, and thermal conductivity. Wei et al. [85] prepared flexible mullite nanofibers via electrospinning based on a nonhydrolytic sol-gel method using anhydrous aluminum chloride (AlCl_3_), TEOS, PVP, and dichloromethane as the main starting materials. The nonhydrolytic sol was prepared first and then fired at 1000 °C for 1 h to obtain flexible mullite nanofibers, and the effect of calcination temperature on the synthesis of mullite fibers was studied. The study indicated that mullite fibers could be synthesized at relatively low heating temperatures. SEM images revealed that when mullite precursors were heated at 800 °C for 1 h, the average fiber diameter decreased from about 395 nm to about 250 nm (Figure 10). This phenomenon may have been induced by the burning-out of PVP after calcination. Meanwhile, the removal of PVP would have also left smooth surfaces on the nanofibers. By increasing the calcination temperature to 1000 °C, the average diameter of fibers was ulteriorly decreased to about 213 nm, which can be ascribed to the complete burn-up of PVP and further shrinkage of the fibers. TEM images of the product fired at 1000 °C for 1 h indicated that the size of the obtained mullite fibers was about 140 nm and the size of mullite grain in fibers was about 20 nm. On the other hand, the digital images of the mullite nanofibers fabricated at 1000 °C for 1 h revealed that the prepared fibers could be easily folded many times without breakage, which illustrated that the as-prepared mullite nanofibers were very soft and flexible. The resultant mullite fiber products that exhibit good high temperature properties could be used in high-temperature industrial and aerospace fields.

**Figure 11:**
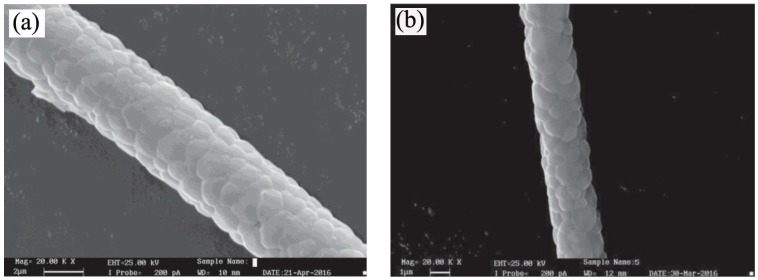
SEM microstructures of (**a**) YAG and (**b**) Cr-YAG precursor gel fibers heated at 1600 °C for 2 h and 6 h, respectively [86].

As the thermodynamically stable yttrium aluminum oxide ceramic, yttrium aluminum garnet (Al_5_Y_3_O_12_ or YAG) has been widely used as high temperature structural materials. Ma et al. [86] prepared chromia-yttrium aluminum garnet (Cr-YAG) long fibers through the sol-gel method using aluminum chloride, aluminum powder, yttrium oxide, chromium trioxide, and acetic acid as the raw materials. The gel fibers were prepared by pulling out the thin glass rod immersed in the spinning sol slowly at room temperature, and the effect of heating temperature on the fabrication of Cr-YAG fibers was investigated. The study indicated that the YAG crystallized directly from the amorphous precursor without forming any intermediate phase. Due to the complete dissolution of Cr_2_O_3_ in solid solution, the Cr_2_O_3_ phase was not detected in the fiber products. SEM images revealed that the grain diameter of YAG and Cr-YAG fibers was about 1.45 and 1.38 nm, respectively (Figure 11), which illustrated that the solid solution ion may affect grain growth by grain growth pinning. The grain growth exponent (*n*) of Cr-YAG fibers (2.88) was slower than that of YAG (*n* = 3), indicating that the grain growth rate was reduced by adding Cr in YAG. 

Composite fibers can also be fabricated by the sol-gel method. Lead magnesium niobate-lead titanate (PMN-PT) single crystals had ultrahigh piezoelectric properties when compared with traditional piezoelectric ceramics. Lam et al. [87] successfully prepared PMN-PT (0.65Pb(Mg_1/3_Nb_2/3_)O_3_-0.35PbTiO_3_) ceramic fibers with the sol-gel method with lead (II) acetate trihydrate, magnesium nitrate salt, niobium(V) ethoxide, and titanium(IV) *n*-butoxide as the main raw materials. The effect of sintering temperature on the fabrication of PMN-PT ceramic fibers was studied, and XRD results revealed that samples sintered at different temperatures (1150, 1200, 1250 °C) had similar perovskite phases. SEM images showed that the microstructure of the PMN-PT fibers was dependent on the sintering temperature, and small cracks were observed in the resultant PMN-PT fibers due to the escaption of the organics during sintering. Interestingly, the relative permittivity and electromechanical coupling coefficient of the prepared PMN-PT ceramic fibers were larger than that of the ceramic disc, thus PMN-PT fibers can be used as reinforcements in 1–3 composites for high-frequency ultrasonic transducer applications.

Aside from the above-mentioned 1D structural materials, other 1D structural materials, including CaZrO_3_ fibers [88], Al_2_O_3_-YAG nanostructured fibers [89], NiO nanofibers [90], Mg_2_Si/CNT thermoelectric nanofibers [91], NbN fibers [92], etc. have also been prepared by the sol-gel technique. With the introduction of the sol-gel technique, 1D structural materials with high quality stoichiometric, uniform diameter, high purity, high homogeneity, and good continuity can be fabricated in large quantity under relatively low temperatures. Furthermore, the morphology (fiber, nanorod, nanowire, and nanotube), shape (hexagonal, round), aspect ratio, density and orientation can be tailored in the sol-gel process. However, cracks and rough surfaces were always observed in the final products. Furthermore, serious shrinkage and loosening structures were usually induced by the elimination of organic impurities. To overcome the existing drawbacks, assisted techniques should be used to decrease the gelling time, and inorganic binders are encouraged for reducing the influence induced by the removal of the organic components.

## 4. Conclusions

As a facile synthesis method, the sol-gel method exhibits some outstanding advantages for fabricating hollow sphere and 1D structural materials such as low reaction temperature, short soaking time, fine particle size, high purity products, and good chemical homogeneity. The as-prepared materials can be used in the fields of high temperature, semiconductor, photoelectric, magnetic, and so on. To extend the use of the sol-gel method, other technologies were introduced such as surface modification, sol coating, organic-inorganic hybridization, templating, etc. More encouragingly, the grain size, size distribution, surface, morphology and homogeneity of the targeted products could be tailored in the process of sol-gel. 

However, long gelling time and various kinds of organic materials were always required to achieve a high quality gel precursor. In the process of removing organic matter, cracks, pores, rough surface, loosened structure and great volume shrinkage may be induced. To improve the quality of the targeted products, the used organics should be removed by calcination, dissolution, or etching. When organic components were burned out, cracks, pores, rough surface and great volume shrinkage may be induced. On the other hand, some of the solvents used to remove the organics were harmful. To enhance the green and environmentally-friendly ability of the sol-gel technique, the amount of volatile solvents and organic additives used in the sol-gel process should be decreased. Furthermore, new processing techniques should be developed to decrease the gelling time and improve the dispersibility of the targeted products. Additionally, the application of the sol-gel technique should be extended from single phase materials to complex systems (binary and ternary system) to fulfill increased demands across various fields. 

We have tried to present the abilities and advantages of the sol-gel method used for preparing hollow sphere and 1D structural materials; however, it was impossible to review all the works carried out on this field. Therefore, only representative investigations were presented in detail with other works listed as references, and we extend our apologies to any overlooked contributions. Although some drawbacks were found in the sol-gel process, there was no doubt that more materials will be prepared by various sol-gel based routes.

## References

[1] Zeynali H., Akbari H. (2016). Magnetic Properties of L1_0_(FePt)_100−x_Ag_x_ Nanoparticles Synthesized by the Sol-Gel Method. J. Supercond. Nov. Magn..

[2] Jang M.S., Roh I.J., Park J.M., Kang C.Y., Choi W.J., Baek S.H., Park S.S., Yoo J.W., Lee K.S. (2017). Dramatic enhancement of the saturation magnetization of a sol-gel synthesized Y_3_Fe_5_O_12_ by a mechanical pressing process. J. Alloys Compd..

[3] Zhang H., Liu Z., Ma C., Yao X., Zhang L., Wu M. (2003). Preparation and microwave properties of Co- and Ti-doped barium ferrite by citrate sol-gel process. Mater. Chem. Phys..

[4] Zhang H., Liu Z., Yao X., Zhang L., Wu M. (2003). Dielectric and magnetic properties of ZnCo-substituted X hexaferrites prepared by citrate sol-gel process. Mater. Res. Bull..

[5] Zhang H., Liu Z., Ma C., Yao X., Zhang L., Wu M. (2002). Complex permittivity, permeability, and microwave absorption of Zn- and Ti-substituted barium ferrite by citrate sol-gel process. Mater. Sci. Eng. B.

[6] Zhang H., Liu Z., Yao X., Zhang L., Wu M. (2003). The Synthesis, Characterization and Microwave Properties of ZnCo-Substituted W-Type Barium Hexaferrite, from a Sol-Gel Precursor. J. Sol-Gel Sci. Technol..

[7] Zhang H., Yao X., Zhang L. (2002). The preparation and microwave properties of BaZn_2−Z_Co_Z_Fe_16_O_27_ ferrite obtained by a sol-gel process. Ceram. Int..

[8] Zhang H., Yao X., Zhang L. (2002). The preparation and microwave properties of Ba_2_Zn_Z_Co_2−Z_Fe_12_O_22_ hexaferrites. J. Eur. Ceram. Soc..

[9] Zhang H., Yao X., Zhang L. (2002). The preparation and microwave properties of Ba_2_Zn_x_Co_2−x_Fe_28_O_46_ hexaferrites. J. Magn. Magn. Mater..

[10] Fernández C.P., Zabotto F.L., Garcia D., Kiminami R.H.G.A. (2017). In Situ sol-gel co-synthesis at as low hydrolysis rate and microwave sintering of PZT/Fe_2_CoO_4_ magnetoelectric composite ceramics. Ceram. Int..

[11] Kahouadji B., Guerbous L., Boukerika A., Dolić S.D., Jovanović D.J., Dramićanin M.D. (2017). Sol gel synthesis and pH effect on the luminescent and structural properties of YPO_4_: Pr^3+^ nanophosphors. Opt. Mater..

[12] Nouri M.S., Kompany A., Khorsand Zak A., Khorrami Gh.H. (2017). Characterization of Ce_(1−x)_Zr_x_O_2_ yellow nanopigments synthesized by a green sol-gel method. Ceram. Int..

[13] Almamoun O., Ma S. (2017). Effect of Mn doping on the structural, morphological and optical properties of SnO_2_ nanoparticles prepared by Sol-gel method. Mater. Lett..

[14] Hu R., Zhao J., Zheng J. (2017). Synthesis of SnO_2_/rGO hybrid materials by sol-gel/thermal reduction method and its application in electrochemical capacitors. Mater. Lett..

[15] Cao E., Wang H., Wang X., Yang Y., Hao W., Sun L., Zhang Y. (2017). Enhanced ethanol sensing performance for chlorine doped nanocrystalline LaFeO_3-δ_ powders by citric sol-gel method. Sens. Actuators B Chem..

[16] Li F., Zhang H., Zhang S., Liang F., Liu J., Cao Y. (2016). Low-temperature preparation of ZrC powders using a combined sol-gel and microwave carbothermal reduction method. J. Ceram. Soc. Jpn..

[17] Zhang H., Li F., Lu L., Zhang S., Cao Y. (2015). Preparation and characterization of ultrafine ZrB_2_-SiC composite powders by a combined sol-gel and microwave boro/carbothermal reduction method. Ceram. Int..

[18] Li F., Fu F., Lu L., Zhang H., Zhang S. (2015). Preparation and artificial neural networks analysis of ultrafine β-Sialon powders by microwave-assisted carbothermal reduction nitridation of sol-gel derived powder precursors. Adv. Powder Technol..

[19] Zhang H., Li F., Jia Q., Ye G. (2008). Preparation of titanium carbide powders by sol-gel and microwave carbothermal reduction methods at low temperature. J. Sol-Gel Sci. Technol..

[20] Zhang H., Li F. (2008). Preparation and microstructure evolution of diboride ultrafine powder by sol-gel and microwave carbothermal reduction method. J. Sol-Gel Sci. Technol..

[21] Zhang H., Wang Z., Zhang H. (2007). Synthesis of O’-SiAlON ultrafine powder. Am. Ceram. Soc. Bull..

[22] Zhang H., Zhang H., Miao J., Wang Z., Jia Q., Jia X. (2007). Preparation of Ultrafine β-Sialon Powder by Citrate Sol-Gel and Carbothermal Reduction Nitridation. Key Eng. Mater..

[23] Zhang H., Yan Y., Liu Z. (2005). Effect of seeds on the synthesis of mullite powder by the citrate sol-gel method. Interceram.

[24] Zhang H., Jia X., Yan Y., Liu Z., Yang D., Li Z. (2004). The effect of the concentration of citric acid and pH values on the preparation of MgAl_2_O_4_ ultrafine powder by citrate sol-gel process. Mater. Res. Bull..

[25] Zhang H., Jia X., Liu Z., Li Z. (2004). The low temperature preparation of nanocrystalline MgAl_2_O_4_ spinel by citrate sol-gel process. Mater. Lett..

[26] Xiao L., Zhao Y., Yin J., Zhang L. (2009). Clewlike ZnV_2_O_4_ hollow spheres: Nonaqueous sol-gel synthesis, formation mechanism, and lithium storage properties. Chem. Eur. J..

[27] Li J., Jiao X., Chen D. (2007). Preparation of Y-TZP ceramic fibers by electrolysis-sol-gel method. J. Mater. Sci..

[28] Yang Q., Sha J., Ma X., Yang D. (2005). Synthesis of NiO nanowires by a sol-gel process. Mater. Lett..

[29] Chelouche A., Touam T., Tazerout M., Djouadi D., Boudjouan F. (2017). Effect of Li codoping on highly oriented sol-gel Ce-doped ZnO thin films properties. J. Lumin..

[30] Chen R., Zhang Y., Liu T., Xu B., Shen Y., Lin Y., Nan C. (2017). Improvement of the conductivity of sol-gel derived Li-La-Zr-O thin films by the addition of surfactant. Ceram. Int..

[31] Ivanova T., Harizanova A., Koutzarova T., Vertruyen B. (2015). Optical characterization of sol-gel ZnO:Al thin films. Superlattice Microstruct..

[32] Kayani Z.N., Riaz S., Naseem S. (2017). Study of Nickel Nitride Thin Films Deposited by Sol-Gel Route. Trans. Indian Inst. Met..

[33] Predoana L., Stanciu I., Anastasescu M., Calderon-Moreno J.M., Stoica M., Preda S., Gartner M., Zaharescu M. (2016). Structure and properties of the V-doped TiO_2_ thin films obtained by sol-gel and microwave-assisted sol-gel method. J. Sol-Gel Sci. Technol..

[34] Ren Q., Zhang Y., Chen Y., Wang G., Dong X., Tang X. (2013). Structure and magnetic properties of La_0.67_Sr_0.33_MnO_3_ thin films prepared by sol-gel method. J. Sol-Gel Sci. Technol..

[35] Guo X., Zhang Q., Ding X., Shen Q., Wu C., Zhang L., Yang H. (2016). Synthesis and application of several sol-gel-derived materials via sol-gel process combining with other technologies: A review. J. Sol-Gel Sci. Technol..

[36] Zhang H., Fu F., Cao Y., Du S., Lu L., Zhang S. (2013). Sol-Gel Process Synthesis of High-Temperature Non-oxide Ultrafine Powders. Interceram.

[37] Livage J., Ganguli D. (2001). Sol-gel electrochromic coatings and devices:A review. Sol. Energy Mater. Sol. Cells.

[38] Yoldas B.E. (1993). Technological significance of Sol-Gel process and process-induced variations in Sol-Gel materials and coatings. J. Sol-Gel Sci. Technol..

[39] Du X., He J. (2009). Facile preparation of titania hollow spheres by combination of the mixed solvent method and the sol-gel process and post-calcination. Mater. Res. Bull..

[40] Dobó D.G., Berkesi D., Kukovecz Á. (2017). Morphology conserving aminopropyl functionalization of hollow silica nanospheres in toluene. J. Mol. Struct..

[41] Dai Z., Meiser F., Möhwald H. (2005). Nanoengineering of iron oxide and iron oxide/silica hollow spheres by sequential layering combined with a sol-gel process. J. Colloid Interf. Sci..

[42] Qiao M., Wu S., Chen Q., Shen J. (2010). Novel triethanolamine assisted sol-gel synthesis of N-doped TiO_2_ hollow spheres. Mater. Lett..

[43] Chen Z., Wang F., Zhang H., Yang T., Cao S., Xu Y., Jiang X. (2015). Synthesis of uniform hollow TiO_2_ and SiO_2_ microspheres via a freezing assisted reverse microemulsion-templated sol-gel method. Mater. Lett..

[44] Teng Z., Han Y., Li J., Yan F., Yang W. (2010). Preparation of hollow mesoporous silica spheres by a sol-gel/emulsion approach. Microporous Mesoporous Mater..

[45] Yin H., Wang X., Wang L., Yuan Q., Zhao H. (2015). Self-doped TiO_2_ hierarchical hollow spheres with enhanced visible-light photocatalytic activity. J. Alloys Compd..

[46] Li X., Zhang D., Chen Y. (2017). Silicone rubber/hollow silica spheres composites with enhanced mechanical and electrical insulating performances. Mater. Lett..

[47] Zhang Y., Li G., Wu Y., Xie T. (2005). Sol-gel synthesis of titania hollow spheres. Mater. Res. Bull..

[48] Fan H., Lei Z., Jia H., Zhao X. (2011). Sol-gel synthesis, microstructure and adsorption properties of hollow silica spheres. Mater. Lett..

[49] Ashuri M., He Q., Zhang K., Emani S., Shaw L.L. (2017). Synthesis of hollow silicon nanospheres encapsulated with a carbon shell through sol-gel coating of polystyrene nanoparticles. J. Sol-Gel Sci. Technol..

[50] Deng W., Chen D., Chen L. (2015). Synthesis of monodisperse CeO_2_ hollow spheres with enhanced photocatalytic activity. Ceram. Int..

[51] Lin X., Rong F., Ji X., Fu D. (2011). Visible light photocatalytic activity and Photoelectrochemical property of Fe-doped TiO_2_ hollow spheres by sol-gel method. J. Sol-Gel Sci. Technol..

[52] Pullar R.C., Taylor M.D., Bhattacharya A.K. (1998). Blow spun strontium zirconate fibers produced from a sol-gel precursor. J. Mater. Sci..

[53] Venkatesh R., Ramanan S.R. (2000). Effect of organic additives on the properties of sol-gel spun alumina fibers. J. Eur. Ceram. Soc..

[54] Chandradass J., Balasubramanian M. (2006). Extrusion of alumina fiber using sol-gel precursor. J. Mater. Sci..

[55] Lee J.H., Kim Y.J. (2014). Hydroxyapatite nanofibers fabricated through electrospinning and sol-gel process. Ceram. Int..

[56] Tan H., Ding Y., Yang J. (2010). Mullite fibers preparation by aqueous sol-gel process and activation energy of mullitization. J. Alloys Compd..

[57] Granger G., Restoin C., Roy P., Jamier R., Roungier S., Lecomte A., Blondy J.M. (2014). Nanostructured optical fibers in the SiO_2_/SnO_2_ system by the sol-gel method. Mater. Lett..

[58] Tan H., Ma X., Fu M. (2013). Preparation of continuous alumina gel fibers by aqueous sol-gel process. Bull. Mater. Sci..

[59] You Y., Zhang S., Wan L., Xu D. (2012). Preparation of continuous TiO_2_ fibers by sol-gel method and its photocatalytic degradation on formaldehyde. Appl. Surf. Sci..

[60] Liu X., Wang J., Zhang J., Yang S. (2007). Sol-gel template synthesis of LiV_3_O_8_ nanowires. J. Mater. Sci..

[61] Senthil T., Anandhan S. (2014). Structure-property relationship of sol-gel electrospun ZnO nanofibers developed for ammonia gas sensing. J. Colloid Interface Sci..

[62] Admaiai L.F., Daza L., Grange P., Delmon B. (1994). Synthesis of YBa_2_Cu_3_O_7−x_ superconductor with fiber structure by the sol-gel method. J. Mater. Sci. Lett..

[63] Boulton J.M., Jones K., Emblem H.G. (1990). The preparation of spinel fiber by a sol-gel route. J. Mater. Sci. Lett..

[64] Ji G., Ji H., Li M., Li X., Sun X. (2014). Synthesis of zirconium diboride nano-powders by novel complex sol-gel technology at low temperature. J. Sol-Gel Sci. Technol..

[65] Cao Y., Du S., Wang J., Zhang H., Li F., Lu L., Zhang S., Deng X. (2014). Preparation of zirconium diboride ultrafine hollow spheres by a combined sol-gel and boro/carbothermal reduction technique. J. Sol-Gel Sci. Technol..

[66] Wang Y., Zhang L., Zhang X., Zhang Z., Tong Y., Li F., Wu J.C.S., Wang X. (2017). Openmouthed β-SiC hollow-sphere with highly photocatalytic activity for reduction of CO_2_ with H_2_O. Appl. Catal. B Environ..

[67] Wang T., Ma W., Shangguan J., Jiang W., Zhong Q. (2014). Controllable synthesis of hollow mesoporous silica spheres and application as support of nano-gold. J. Solid State Chem..

[68] Zhang L., Luo J., Wu M., Jiu H., Chen Q. (2007). Synthesis of Eu_2_O_3_ hollow submicrometer spheres through a sol-gel template approach. Mater. Lett..

[69] Syoufian A., Manako Y., Nakashima K. (2015). Sol-gel preparation of photoactive srilankite-type zirconium titanate hollow spheres by templating sulfonated polystyrene latex particles. Powder Technol..

[70] Yang X., Chaki T.K. (1996). Millimetre-sized hollow spheres of lead zirconate titanate by a sol-gel method. J. Mater. Sci..

[71] Hu Q., Li Y., Zhao N., Ning C., Chen X. (2014). Facile synthesis of hollow mesoporous bioactive glass sub-micron spheres with a tunable cavity size. Mater. Lett..

[72] Toyama N., Ohki S., Tansho S., Shimizu T., Umegaki T., Kojima Y. (2017). Influence of alcohol solvents on morphology of hollow silica–alumina composite spheres and their activity for hydrolytic dehydrogenation of ammonia borane. J. Sol-Gel Sci. Technol..

[73] Zhu Z., Kao C.T., Tang B., Chang W., Wu R. (2016). Efficient hydrogen production by photocatalytic water-splitting using Pt-doped TiO_2_ hollow spheres under visible light. Ceram. Int..

[74] Lu H.T., Tseng I.H. (2015). Fabrication of organosilica hollow spheres using organosiloxane-templated sol-gel process. J. Sol-Gel Sci. Technol..

[75] Mkhalid I.A., Abdulsalam A.A. (2015). Photocatalytic reduction of Hg using core-shell Fe/CeO_2_ hollow sphere nanocomposites. Ceram. Int..

[76] Katagiri K., Kamiya J., Koumoto K., Inumaru K. (2012). Preparation of hollow titania and strontium titanate spheres using sol-gel derived silica gel particles as templates. J. Sol-Gel Sci. Technol..

[77] Chronakis I.S. (2005). Novel nanocomposites and nanoceramics based on polymer nanofibers using electrospinning process-A review. J. Mater. Process. Technol..

[78] Nagamatsu J., Nakagawa N., Muranaka T., Zenitani Y., Akimitsu J. (2001). Superconductivity at 39 K in magnesium diboride. Nature.

[79] Nath M., Parkinson B.A. (2006). A Simple Sol-Gel Synthesis of Superconducting MgB_2_ Nanowires. Adv. Mater..

[80] Li J., Zhang Y., Li G. (2012). Preparation and characterization of SiBON fiber. Mater. Lett..

[81] Tang W., Shan X., Li S., Liu H., Wu X., Chen Y. (2014). Sol-gel process for the synthesis of ultrafine MnO_2_ nanowires and nanorods. Mater. Lett..

[82] Zhou G., Kang Y.S. (2004). Synthesis and structural properties of manganese titanate MnTiO_3_ nanoparticle. Mater. Sci. Eng. C.

[83] Nakhowong R. (2015). Fabrication and characterization of MnTiO_3_ nanofibers by sol-gel assisted electrospinning. Mater. Lett..

[84] Shamitha C., Senthil T., Wu L., Kumar B.S., Anandhan S. (2017). Sol-gel electrospun mesoporous ZnMn_2_O_4_ nanofibers with superior specific surface area. J. Mater. Sci. Mater. Electron..

[85] Wei H., Li H., Cui Y., Sang R., Wang H., Wang P., Bu J., Dong G. (2017). Synthesis of flexible mullite nanofibers by electrospinning based on nonhydrolytic sol-gel method. J. Sol-Gel Sci. Technol..

[86] Ma X., Lv Z., Tan H., Nan J., Wang C., Wang X. (2017). Preparation and grain-growth of chromia-yttrium aluminum garnet composites fibers by sol-gel method. J. Sol-Gel Sci. Technol..

[87] Lam K.H., Li K., Chan H.L.W. (2005). Lead magnesium niobate-lead titanate fibers by a modified sol-gel method. Mater. Res. Bull..

[88] Liu B., Lin X., Zhu L., Wang X., Xu D. (2014). Fabrication of calcium zirconate fibers by the sol-gel method. Ceram. Int..

[89] Shojaie-Bahaabad M., Taheri-Nassaj E., Naghizadeh R. (2008). An alumina-YAG nanostructured fiber prepared from an aqueous sol-gel precursor: Preparation, rheological behavior and spinnability. Ceram. Int..

[90] George G., Anandhan S. (2015). Comparison of structural, spectral and magnetic properties of NiO nanofibers obtained by sol-gel electrospinning from two different polymeric binders. Mater. Sci. Semicond. Process..

[91] Kikuchi K., Yamamoto K., Nomura N., Kawasaki A. (2017). Synthesis of n-type Mg_2_Si/CNT Thermoelectric Nanofibers. Nanoscale Res. Lett..

[92] Nomura K., Takasuka Y., Kamiya K., Nasu H. (1994). Preparation of NbN fibers by nitridation of sol-gel derived Nb_2_O_5_ fibers. J. Mater. Sci. Mater. Electron..

